# A ‘fish finder’ for birds? Route-dependent foraging behavior of the brown booby (*Sula leucogaster*) following a passenger ferry in the Ogasawara Islands, Japan

**DOI:** 10.7717/peerj.21093

**Published:** 2026-05-08

**Authors:** Ryota Hayashi

**Affiliations:** Research & Development Center, Nippon Koei Co., Ltd., Tsukuba, Ibaraki, Japan

**Keywords:** Seabird behavior, Foraging ecology, Vessel-following, *Sula leucogaster*, Ogasawara Islands, Citizen science, Opportunistic foraging

## Abstract

While seabird associations with fishing vessels are well-documented, interactions with non-fishing vessels remain poorly understood. This study investigated the foraging behavior of the brown booby (*Sula leucogaster*) associated with a passenger ferry in the Ogasawara Islands, Japan, home to one of the largest breeding colonies in the archipelago. During four daytime, two-hour trips aboard the inter-island ferry Hahajima-Maru in 2017, the number of accompanying boobies and their plunge-diving events were recorded once per minute. A consistent directional asymmetry was observed in this behavior. Trips originating from the main colony island (Hahajima) showed substantially higher numbers of accompanying birds and dives compared to southbound trips. Birds followed the ferry immediately upon departure from Hahajima but only appeared mid-route when traveling towards the colony. This activity was highest in the August observation, coinciding with the chick-rearing period. These observations suggest that the ferry may function as a ‘fish finder,’ disturbing surface-associated prey (*e.g.*, flying fish) that boobies exploit by diving. The spatially structured pattern, influenced by colony proximity, indicates potential cognitive abilities such as spatial memory and associative learning. This study demonstrates that simple, low-cost observational methods from public transport can yield valuable behavioral insights and offer a scalable approach for citizen science.

## Introduction

“*Whales are found in the sea, also huge crawfish, enormous shells, and echini, which are called ‘gall of the sea. The ocean here is unusually rich in various products.*”

Hawks (1856)

The Ogasawara Islands have long been recognized for their rich marine biodiversity as noted by Commodore Perry’s expedition in Hawks (1856), a characterization that remains apt today. As I finished my 24-hour voyage on the liner *Ogasawara-Maru* from Tokyo and transferred to the *Hahajima-Maru* at Chichijima Island to head to Hahajima Island, I noticed large, conspicuous birds accompanying the ferry throughout the cruise.

While seabird associations with vessels have been extensively documented, particularly in the context of commercial fishing operations that provide scavenging opportunities (Cherel, Weimerskirch & Duhamel, 1996; Votier et al., 2010; Yorio et al., 2010), comparable interactions with non-fishing vessels (and other vehicles) have received far less attention, and reports are relatively sparse and largely anecdotal (*e.g.*, Cimino et al., 2025).

The brown booby (*Sula leucogaster*) is a tropical and subtropical seabird known for its spectacular plunge-diving foraging behavior (Yoda, Kohno & Naito, 2004). In the Ogasawara Islands (also known as the Bonin Islands), a subtropical oceanic archipelago located approximately 1,000 km south of mainland Japan, the brown booby is one of the most frequently observed seabird species in this region (Ozawa & Saotome, 1968; Saotome, 1968). The breeding distribution of brown boobies in this archipelago is highly asymmetric: major colonies are concentrated on uninhabited islets surrounding Hahajima Island (Chiba et al., 2007; Kawaguchi, Mukou & Katsube, 2014), while Chichijima Island, the administrative center, hosts very few breeding individuals due to habitat loss and introduced predators such as feral cats (Horikoshi, 2007; Kawakami & Masuko, 2008).

Despite this asymmetric distribution, brown boobies are frequently observed following the regular inter-island passenger ferry *Hahajima-Maru*, which travels once or twice daily between Chichijima and Hahajima.

**Figure 1:**
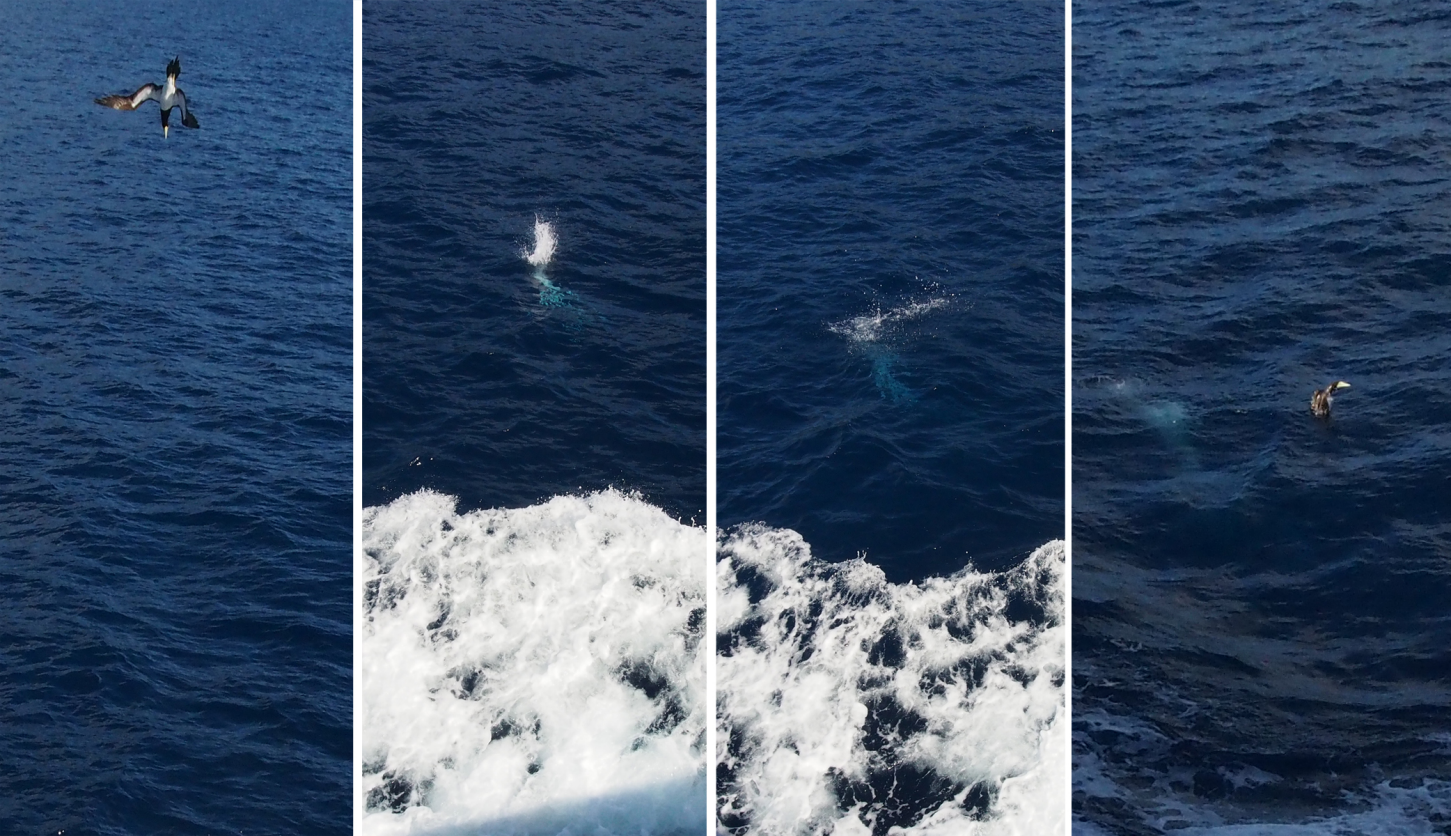
Feeding dive of a brown booby (*Sula leucogaster*) photographed from the upper deck of the inter-island ferry *Hahajima-Maru* near Hahajima Island, Ogasawara Islands, Japan. Photograph by Ryota Hayashi.

This study documents the ferry-following and diving behaviors of brown boobies in the Ogasawara Islands through systematic visual observations (Fig. 1; Supplementary material S1). This study demonstrates that meaningful behavioral data can be collected using simple, low-cost methods accessible to citizen scientists, offering a scalable approach for monitoring seabird behavior in remote regions. Understanding such patterns could provide insights into seabird cognitive abilities, foraging ecology, and human-wildlife interactions in marine environments.

## Materials and Methods

The study was conducted in the Ogasawara Islands, a subtropical oceanic archipelago located approximately 1,000 km south of mainland Japan. The inter-island ferry *Hahajima-Maru* operates regular service between Chichijima Island (27°05′N, 142°11′E) and Hahajima Island (26°38′N, 142°09′E), with each one-way trip taking approximately two hours and covering a distance of approximately 50 km. During transit, flying fish (*Cypselurus* spp.) were frequently observed to be disturbed near the surface around the vessel.

Brown boobies in the Ogasawara Islands breed primarily on uninhabited islets surrounding Hahajima Island (Chiba et al., 2007; Kawaguchi, Mukou & Katsube, 2014). The breeding season extends from spring through summer. August corresponds to the peak of chick-rearing activity when parental provisioning is intensive (Momiyama, 1930). Chicks fledge approximately three months after hatching (Ospina-Alvarez, 2014).

Visual observations were conducted during four daytime ferry trips in 2017: April 9, May 12, August 3, and August 10 (Table 1). Two trips were southbound (Chichijima to Hahajima: April 9 and August 3) and two were return trips (northbound, Hahajima to Chichijima: May 12 and August 10). All observations were made under clear or partly cloudy conditions with good visibility.

**Table 1:** Summary of the four daytime ferry cruises surveyed in 2017 in the Ogasawara Islands, Japan, showing date, time, travel direction, cumulative number of accompanying brown boobies (sum of 1-min counts), and number of dives.

Date	Time	Bound	Individuals	Dives
2017. 4. 9.	12:00-14:00	Southbound	108	5
2017. 5. 12.	12:00-14:00	Northbound	431	71
2017. 8. 3.	14:00-16:00	Southbound	193	59
2017. 8. 10.	10:00-12:00	Northbound	474	113

Observations were conducted by a single observer (RH) from both the lower and upper decks of the ferry to maximize the field of view. The number of accompanying brown boobies flying ahead of, alongside, or behind the vessel within visible range (approximately within 200 m of the vessel; estimated), and the number of diving events were recorded once per minute using a stopwatch and field notebook. Each observation session began immediately after departure and continued until the ferry reached its destination. Because the number of accompanying brown boobies was low (typically ≤10 individuals at any moment), all birds and diving events could be visually monitored by a single observer. The observer continuously adjusted position on the upper deck to maintain a clear view of the vessel’s surroundings.

GPS tracks of each cruise were recorded using the Runkeeper mobile app (FitnessKeeper Inc., USA) on an Android smartphone to confirm cruise duration, route, and direction. The total number of accompanying individuals and dives were summed for each cruise to compare between outbound and return trips.

Portions of this text were previously published as part of a preprint (Hayashi, 2025).

## Results

**Figure 2:**
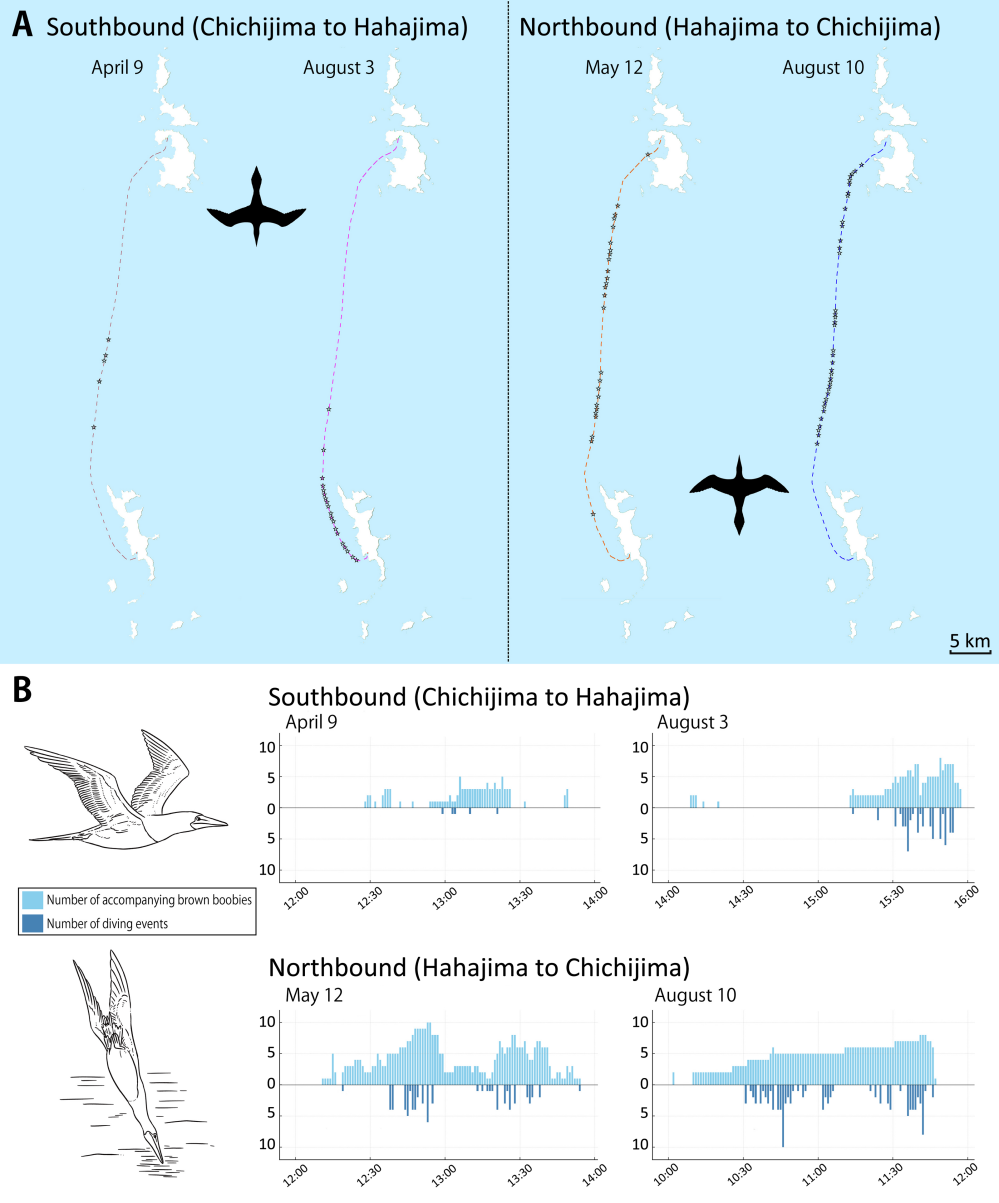
Route-dependent foraging behavior of brown boobies observed from the inter-island ferry *Hahajima-Maru*. (A) Map showing the ferry routes for the four cruises. Silhouettes indicate the direction of travel for southbound (Chichijima to Hahajima) and northbound (Hahajima to Chichijima) cruises. Stars mark the locations of feeding dive events. (B) Frequency distribution of the number of accompanying brown boobies (upper panel) and feeding dive events (lower panel) for each cruise, plotted along a timeline from departure to arrival. Booby flying and diving illustrations by Dr. Chihiro Kinoshita, used with permission.

Brown boobies were observed accompanying the ferry on all four cruise trips. However, a consistent directional pattern was evident in both the number of individuals and the frequency of dives (Fig. 2).

On southbound cruises from Chichijima to Hahajima (April 9 and August 3), brown boobies were rarely present during the first half of the route. Birds began to appear approximately halfway through the journey and increased in number as the vessel approached Hahajima. In contrast, on northbound cruises from Hahajima to Chichijima (May 12 and August 10), brown boobies were already present immediately after departure and remained active throughout the route.

Consequently, the cumulative number of accompanying individuals and dives were substantially higher on northbound trips compared to southbound trips (Table 1). Seasonal variation was also observed, with markedly greater activity recorded in August compared to spring (April and May). This elevated activity in August coincides with the peak chick-rearing period for brown boobies in the Ogasawara Islands (Momiyama, 1930).

## Discussion

This study provides the first documentation of route-dependent foraging-related behavior in brown boobies following a passenger ferry, but it has several limitations. The small sample size (*n* = 4 cruises) precludes formal statistical testing; however, the directional contrast was consistent across dates and in both travel directions, suggesting that the pattern is biologically meaningful. Observations were conducted by a single observer, which may introduce observer bias and imperfect detection of all birds and plunge-diving events. In addition, birds were not individually identified, and observations were not focused on single individuals; therefore, it cannot be determined whether repeated dives were performed by the same individuals or by different birds. Weather conditions also varied slightly among cruises, potentially affecting bird activity and detectability.

Despite these limitations, the consistent directional asymmetry—with substantially more accompanying birds and diving events on routes departing from the nesting-rich Hahajima Island—suggests that the ferry may function as a “fish finder” for brown boobies. The vessel likely disturbs surface-associated prey, which birds may exploit through feeding. Seabirds often rely on visual cues—such as the behavior of other predators or the presence of vessels—to locate ephemeral prey patches (Monier, 2024; Collet et al., 2025).

This phenomenon represents a remarkable role reversal from the species’ traditional ecology. Brown boobies are known in Japanese as “*Katsuo-Dori*” (skipjack bird) because they historically followed schools of skipjack tuna (*Katsuwonus* spp.) that drive prey to the surface—a natural fish finder used by fishermen. In the Ogasawara Islands, these birds now appear to use the ferry as their own fish finder, demonstrating behavioral flexibility and opportunistic foraging strategies.

The spatially structured pattern observed in this study suggests that colony proximity influences foraging behavior. Birds were immediately present on departure from Hahajima (where breeding colonies are concentrated) but appeared only around the midpoint on southbound routes from Chichijima, where few birds breed as reported in Saotome (1968). This pattern points to the possibility that brown boobies may possess sophisticated cognitive abilities, including spatial memory of colony locations and associative learning linking the ferry with foraging opportunities. Furthermore, the consistency of the vessel’s timing suggests that birds may utilize temporal memory to anticipate these foraging interactions, adding a cognitive dimension to this opportunistic behavior.

The delayed appearance of birds on southbound routes is consistent with the detection range documented in the closely related northern gannet (*Morus bassanus*), which can locate fishing vessels up to 11 kilometers away (Bodey et al., 2014). Brown boobies around Hahajima may not detect the ferry until it reaches approximately the halfway point, at which distance they can intercept it for the remainder of the journey.

The elevated foraging activity observed in August, coinciding with the peak chick-rearing period (Momiyama, 1930), suggests that parental birds may rely more heavily on this predictable food source when provisioning offspring. Brown booby chicks require intensive parental care for approximately three months from hatching to fledging (Ospina-Alvarez, 2014), creating substantial energetic demands on parents. Seabirds usually forage further away from the colony during incubation than chick rearing (Ito et al., 2010; Robertson et al., 2014). Given this, the regular, predictable schedule of the ferry (once or twice daily) may represent a reliable supplementary foraging opportunity during this critical period.

This discovery raises numerous questions about the mechanisms underlying this behavior. What type of individuals exploit the ferry? Are they subordinate individuals outcompeted during prime foraging times, or innovative individuals that have discovered an alternative foraging strategy? How is this behavior transmitted—through cultural learning from parent to offspring, or through other social learning mechanisms? Does individual variation exist in ferry-following propensity, and does exploitation of this resource translate into measurable fitness benefits? The consistent, spatially structured pattern documented here suggests potential tool use or proto-tool use behavior, where the ferry serves as an external object that extends the birds’ foraging capabilities (Seed & Byrne, 2010). Future studies integrating bio-logging technologies such as GPS trackers, accelerometers, and animal-borne video loggers (Watanabe & Papastamatiou, 2023; Sato et al., 2025) could identify which individuals use the ferry, quantify their foraging success relative to non-users, and reveal the cognitive mechanisms and learning processes underlying this adaptation. Unfortunately, I did not quantify prey availability or ferry-induced disturbance of flying fish, so the mechanism linking ferry passage to booby foraging remains inferential. Direct observations or video documentation of prey disturbance events would provide stronger evidence for the “fish finder” hypothesis.

Importantly, this study demonstrates that meaningful behavioral data can be collected using simple, low-cost methods requiring no specialized equipment. The observations reported here were made using only a pen, notebook, stopwatch, and smartphone GPS app. This accessible approach is easily replicable and scalable, offering a powerful tool for ecological monitoring, particularly in remote regions like the Ogasawara Islands. The Ogasawara archipelago is both attractive to tourists (28,651 visitors in 2024; 21,435 to Chichijima and 7,216 to Hahajima; Tokyo Metropolitan Government, 2025) and extremely remote from mainland Japan (24 h by ferry from Tokyo, plus 2 additional hours to Hahajima). This remoteness limits scientific access by professional researchers, but creates opportunities for citizen science. Tourists using the *Hahajima-Maru* could easily conduct visual surveys using the methods described here.

While most citizen science projects follow a top-down, researcher-driven “Contributory model”, the method proposed here enables bottom-up, “Co-created model” research that begins with the curiosity and discoveries of citizens themselves (Haklay, 2012; Shirk et al., 2012). If survey results by tourists accumulate over time, they could contribute significantly to advances in animal ecology and cognition. This case study suggests that anyone, even non-experts, can contribute to scientific discovery with simple tools and careful observation. Future studies with larger sample sizes, multiple observers, and standardized protocols would strengthen these findings.

To facilitate transparent data accumulation, a standardized fieldnote template is provided (Supplementary material S2). In the longer term, such records could be mobilized through institutional biodiversity repositories to ensure long-term accessibility and governance. Contributors who wish to make their raw records citable can additionally archive datasets in open repositories (*e.g.*, Zenodo or OSF) under their own authorship (as done in this study; see Hayashi, 2026). To ensure discoverability, I suggest tagging the dataset with the keyword “Ogasawara_Booby_Survey”. This ensures that data remain accessible, citable, and governed by the contributors themselves, while remaining easily searchable for future meta-analyses. As a fallback, contributors may contact the corresponding author to discuss potential collaboration or secondary analyses; however, routine data sharing is best handled *via* public repositories under the contributor’s own authorship.

## Conclusion

This study documents route-dependent foraging behavior in brown boobies following a passenger ferry in the Ogasawara Islands, Japan. The consistent directional asymmetry and spatial structuring of foraging activity suggest that the ferry functions as a “fish finder”, and that birds possess cognitive abilities including spatial memory and associative learning. Elevated activity during the breeding season indicates that this predictable resource may be particularly important for provisioning offspring. This work demonstrates the value of simple, low-cost observational methods accessible to citizen scientists for advancing understanding of seabird behavior and ecology in remote marine environments.

##  Supplemental Information

10.7717/peerj.21093/supp-1 Supplemental Information 1 Fieldnote template An example of the fieldnote template for recording the foraging behavior of brown boobies as conducted in this study. Designed to be printed double-sided on a single sheet, this standardized format can be used as a ready-to-use tool for future monitoring by citizen scientists and tourists.

10.7717/peerj.21093/supp-2 Supplemental Information 2 Plunge diving behavior of brown boobies (*Sula leucogaster* ) observed from the deck of the inter-island ferry *Hahajima-Maru* Supplementary Material S1. Plunge diving behavior of brown boobies (*Sula leucogaster*) observed from the deck of the inter-island ferry *Hahajima-Maru* near Hahajima Island, Ogasawara Islands, Japan. This video captures a typical dive (plunge dive) performed by a brown booby in close proximity to the ferry. The footage demonstrates that such foraging behavior is clearly visible to the naked eye from the passenger deck, highlighting the feasibility of visual surveys without specialized equipment. These observations support the study’s claim that vessel-following behavior can be effectively monitored by citizen scientists aboard regular ferry routes.

## Additional Information and Declarations

### Data Availability

The following information was supplied regarding data availability: The data is available at Zenodo: Hayashi, R. (2026). Dataset for: Route-dependent foraging behavior of the brown booby (Sula leucogaster) following a passenger ferry in the Ogasawara Islands, Japan [Data set]. Zenodo. https://doi.org/10.5281/zenodo.18442896.
