# Supplementary material for: A ‘fish finder’ for birds? Route-dependent foraging behavior of the brown booby (Sula leucogaster) following a passenger ferry in the Ogasawara Islands, Japan
Source: PeerJ. 2026 May 8;14:e21093. doi: 10.7717/peerj.21093 (PMC13159731; doi:10.7717/peerj.21093)
Supplement: Supplemental Information 1 — An example of the fieldnote template for recording the foraging behavior of brown boobies as conducted in this study. Designed to be printed double-sided on a single sheet, this standardized format can be used as a ready-to-use tool for future monitoring by citizen scientists and tourists. [file peerj-14-21093-s001.pdf]

Date: Weather condition: From: To: Recorded by

**Minute from departure**	**Number of accompanying brown boobies** 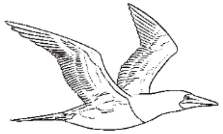	**Number of plunge-diving events** 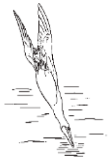	**Minute from departure**	**Number of accompanying brown boobies** 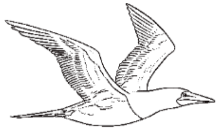	**Number of plunge-diving events** 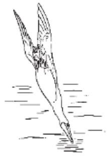
1			31		
2			32		
3			33		
4			34		
5			35		
6			36		
7			37		
8			38		
9			39		
10			40		
11			41		
12			42		
13			43		
14			44		
15			45		
16			46		
17			47		
18			48		
19			49		
20			50		
21			51		
22			52		
23			53		
24			54		
25			55		
26			56		
27			57		
28			58		
29			59		
30			60		

* Note: How to use this sheet
1. Observations were recorded at 1-minute intervals from departure to arrival (total 120 minutes).
2. The number of accompanying birds represents the maximum number of individuals visible around the vessel during each minute.
3. Plunge-diving events were counted whenever a dive was visually confirmed during the same minute.
4. Counts were recorded manually using tally marks while continuously observing from the upper deck.

Supplementary material S2 for: Hayashi R. 2026. A `fish finder' for birds? Route-dependent foraging behavior of the brown booby (*Sula leucogaster*) following a passenger ferry in the Ogasawara Islands, Japan. *PeerJ* 14:e21093 http://doi.org/10.7717/peerj.21093

Illustrations of brown booby: © Chihiro Kinoshita. Used with permission.

Date: Weather condition: From: To: Recorded by

Minute from departure	Number of accompanying brown boobies 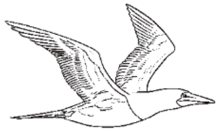	Number of plunge-diving events 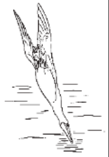	Minute from departure	Number of accompanying brown boobies 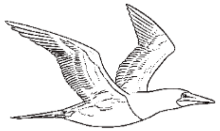	Number of plunge-diving events 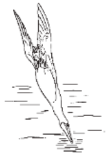
61			91		
62			92		
63			93		
64			94		
65			95		
66			96		
67			97		
68			98		
69			99		
70			100		
71			101		
72			102		
73			103		
74			104		
75			105		
76			106		
77			107		
78			108		
79			109		
80			110		
81			111		
82			112		
83			113		
84			114		
85			115		
86			116		
87			117		
88			118		
89			119		
90			120		

* Note: How to share your record (optional)

1. Archive your completed fieldnote and any raw data under your own authorship (e.g., Zenodo) and obtain a DOI.
2. If you post photos/videos, include a brief caption (date, ferry route, time window).
3. For questions about archiving or potential collaboration (reply not guaranteed), see the corresponding author details in the associated article, or contact: bubobubo32[at]gmail.com.

Supplementary material S2 for: Hayashi R. 2026. A `fish finder' for birds? Route-dependent foraging behavior of the brown booby (*Sula leucogaster*) following a passenger ferry in the Ogasawara Islands, Japan. *PeerJ* 14:e21093 http://doi.org/10.7717/peerj.21093

Illustrations of brown booby: © Chihiro Kinoshita. Used with permission.
